# Tau positron emission tomography analysis methods for the quantification of tau spread in preclinical and early Alzheimer's disease

**DOI:** 10.1002/alz.71573

**Published:** 2026-06-23

**Authors:** Janice Wong, Tina Wang, Ritobrato Datta, Rouhollah O. Abdollahi, Jingwei Li, Christopher C Rowe, David Henley, Hartmuth C. Kolb, Ziad S. Saad, for the Alzheimer's Disease Neuroimaging Initiative

**Affiliations:** ^1^ Johnson & Johnson Cambridge Massachusetts USA; ^2^ Johnson & Johnson La Jolla California USA; ^3^ Johnson & Johnson Leiden The Netherlands; ^4^ Johnson & Johnson Neuss Germany; ^5^ Department of Molecular Imaging & Therapy Austin Health Heidelberg Victoria Australia; ^6^ The Florey Institute of Neuroscience and Mental Health The University of Melbourne Parkville Victoria Australia; ^7^ Johnson & Johnson Titusville New Jersey USA; ^8^ Johnson & Johnson La Jolla California USA

**Keywords:** Alzheimer's disease, biomarker, flortaucipir, mild cognitive impairment, MK‐6240 (florquinitau), preclinical Alzheimer's disease, region of interest, spatial progression of tauopathy, standardized uptake value ratio, tau PET, tau positron emission tomography

## Abstract

**INTRODUCTION:**

Inhibition of pathological tau spread may slow cognitive decline in Alzheimer's disease. Here, we evaluate two tau positron emission tomography (PET) analysis methods designed for detecting tau spread.

**METHODS:**

Spatial progression of tauopathy (SPOT) and tau‐naïve anatomical region of interest (tau‐naïve ROI) methods were assessed in two large observational cohorts in amyloid‐positive (A+) cognitively unimpaired (CU) or mildly cognitively impaired (MCI) participants versus CU amyloid‐negative (A−) participants.

**RESULTS:**

SPOT and tau‐naïve ROI demonstrated measurable annualized change in CU A+ and MCI A+ participants versus CU A− participants. These spread estimates produced effect sizes comparable to standardized uptake value ratio (SUVR) change measures in anatomically defined ROIs. Effect sizes were increased when evaluating the subset of tau positive CU A+ and MCI A+ participants.

**DISCUSSION:**

SPOT and tau‐naïve ROI methods are designed to measure tau spread and may be used in addition to traditional SUVR measures to evaluate tau progression in AD.

## Highlights

- SPOT and tau‐naïve ROI are two methods of measuring tau spread in vivo using PET.
- SPOT and tau‐naïve ROI detected tau spread in A+ participants.
- SPOT and tau‐naïve ROI may be useful for evaluating drugs targeting AD tau spread.

## 1 BACKGROUND

Abnormal accumulation of intracellular neurofibrillary tangles (NFTs) of pathological tau is a defining hallmark of Alzheimer's disease (AD). NFTs can appear several years prior to onset of overt signs and symptoms of AD and spread in distinct spatiotemporal patterns in the brain as AD progresses.^1^, ^2^ It is hypothesized that pathological tau spreads between brain regions through tau seeding, whereby tau seeds – single molecules or oligomers of phosphorylated tau released from NFTs in degenerating neurons – propagate to unaffected cells and induce NFT formation.^3^, ^4^, ^5^ Spreading may be the predominant method of new tau accumulation prior to Braak stage 3, whereas local accumulation via replication may dominate in later stages.^6^ Consistent with this hypothesis, non‐clinical models have shown that extracellular tau seeds can induce neuronal NFT accumulation.^7^ In human AD brains, tau oligomers are found in higher proportions than phosphorylated or misfolded tau in synaptic terminals, even in areas that do not yet have substantial NFT accumulation.^8^ Abnormal tau spread via tau seeds may be mediated by functionally connected networks^9^, ^10^, ^11^, ^12^ and through interactions with amyloid beta (Aβ).^12^, ^13^ Thus, tau spread may be a promising target for investigational therapies in AD.

Recent approvals of amyloid‐based therapies in early symptomatic AD have established precedent for accelerated approval of AD medications based on positron emission tomography (PET)‐based surrogate endpoints. Data from aducanemab, lecanemab, and donanemab studies^14^, ^15^, ^16^ demonstrate that treatments that reduce amyloid PET signals may slow clinical AD progression. Compared to amyloid pathology, accumulation of cortical tau pathology is more strongly correlated with clinical AD progression than Aβ plaques,^17^, ^18^ as demonstrated in multiple PET imaging‐based studies.^19^, ^20^, ^21^, ^22^, ^23^ Both baseline tau burden and regional patterns of change in tau PET are associated with cognitive outcomes.^24^, ^25^, ^26^ Given the close association between pathological tau and clinical decline, it is possible that tau‐related biomarkers could be similarly developed as surrogate biomarkers for clinical progression in AD, if future data with tau therapies are supportive.

Many of the current anti‐tau therapies under investigation for AD are hypothesized to slow cognitive decline by inhibiting the spread of pathological tau seeds.^27^ The common approach of assessing tau PET spread with standardized uptake value ratio (SUVR) change measures across anatomically defined regions of interest (ROIs) may not be sensitive enough to detect such anti‐tau treatment effects. To enable detection of the treatment effects of therapies purported to slow tau spread, measures designed for quantifying tau spread may be useful as complementary methods.

The objective of this study was to describe and evaluate two tau PET analysis methods that have been designed to measure tau spread. We evaluated their ability to detect tau PET progression using observational longitudinal data from multiple clinical cohorts that used different tau PET tracers. The first method is spatial progression of tauopathy (SPOT), in which the spread estimate is measured by subtracting the fraction of cortex with high uptake at the follow‐up visit from that at the baseline visit. This method is consistent with published methods for measuring tau spatial spread (TSS), such as Spatial Extent of Tau measures.^19^, ^24^, ^28^, ^29^, ^30^, ^31^, ^32^ The second method is a novel approach, in which change in tau PET SUVR is assessed over a ROI consisting of voxels without NFT evidence at baseline. This ROI – termed tau‐naïve ROI – is an individual‐specific composite consisting of the aggregate of cortical voxels where baseline SUVR is within 1 standard deviation (SD) of the average SUVR in cognitively unimpaired (CU) amyloid‐negative (A−) cohorts. We demonstrate the performance of the SPOT and tau‐naïve ROI methods in longitudinal cohorts and discuss their potential to be used in addition to traditional SUVR measures to evaluate tau progression.

## 2 METHODS

### 2.1 Participants

Participants forming the longitudinal cohorts in this study were selected from the Australian Imaging, Biomarkers and Lifestyle Flagship Study of Ageing (AIBL) (https://aibl.org.au/), the Cerveau Technologies database (Cerveau) (https://www.cerveautechnologies.com/science/), and the Alzheimer's Disease Neuroimaging Initiative (ADNI) database (adni.loni.usc.edu). The ADNI was launched in 2003 as a public–private partnership, led by Principal Investigator Michael W. Weiner, MD. The primary goal of ADNI has been to test whether serial magnetic resonance imaging (MRI), PET, other biological markers, and clinical and neuropsychological assessment can be combined to measure the progression of mild cognitive impairment (MCI) and early AD.

**RESEARCH IN CONTEXT**

1. **Systematic review**: Abnormal accumulation of pathological intracellular tau NFTs is a defining hallmark of AD. According to the tau spread hypothesis, pathological tau propagates from affected to unaffected cells via tau seeds.
2. **Interpretation**: Two tau PET methods designed to measure tau spread – SPOT and tau‐naïve ROI – were evaluated in two large observational cohorts comparing A+ CU or MCI participants versus CU A− participants. Both methods demonstrated measurable annualized change in CU and MCI A+ participants versus CU A− participants, with increased effect sizes observed in tau PET‐positive CU and MCI A+ participants.
3. **Future directions**: SPOT and tau‐naïve ROI methods are designed to measure tau spread and may be useful in addition to traditional SUVR measures to evaluate pharmacological interventions targeting tau progression in AD.

As ADNI participants underwent tau PET imaging using AV1451 (^18^F‐T807, ^18^F‐flortaucipir, ^18^F‐FTP),^33^ this cohort was designated as the Flortaucipir cohort. Similarly, as AIBL and Cerveau participants underwent tau PET imaging using ^18^F‐MK‐6240 (^18^F‐florquinitau), this cohort was designated as the MK‐6240 cohort. Participants were selected if they had a baseline tau PET scan and T1‐weighted (T1) structural MRI and had at least one follow‐up tau PET scan at least 9 months after the initial tau PET scan. Age, sex, education, apolipoprotein E (APOE) status, Mini‐Mental State Examination (MMSE),^34^ and Clinical Dementia Rating Global Score (CDR GS) of participants at baseline (defined as the participant's first tau PET visit) were also obtained from these databases, if available. In addition to these longitudinal cohorts, we used cross‐sectional cohorts of CU A− participants from the aforementioned Cerveau, AIBL, and ADNI cohorts to derive control distributions of SUVR values in the absence of AD pathology.

Participants from the longitudinal cohorts were grouped based on cognitive and amyloid status: CU amyloid positive (A+), MCI A+, and healthy control (CU A−) groups, using definitions of CU and MCI as previously described.^35^, ^36^ Specifically, participants from AIBL were determined to be CU if within 1.5 SD of published normative data for their age group based on a battery of cognitive tests, and MCI was determined according to criteria by Winblad et al. and Petersen et al.^34^, ^37^, ^38^, ^39^, ^40^ In the ADNI study, clinical characterization was based on memory complaints (CU participants had none, while MCI participants had memory complaints); CDR score (0 for CU and 0.5 [with memory box score ≥0.5] for MCI); and delayed recall from the Logical Memory II subscale of the Wechsler Memory Scale‐Revised.^36^, ^38^ Furthermore, subgroup analyses were performed for CU and MCI A+ participants who were also tau positive (T+). Tau positive was defined as having a z‐score ≥1 relative to the tracer matched cross sectional control cohort in the inferior temporal lobe ROI.

#### 2.1.1 Tracer‐specific control cohorts

To create tracer‐specific normative distributions of tau PET SUVR, we analyzed scans of CU A− participants that were available at the time of method development. This tracer‐specific control cohort included baseline tau PET scans from CU A− participants who had only one available tau PET scan, as well as from CU A− participants with longitudinal tau PET data (Table 1).

**TABLE 1:** Baseline demographic and clinical characteristics of participant groups in Flortaucipir and MK‐6240 cohorts.

Flortaucipir cohort (*N* = 221)	Control (CU A−) (*n* = 100)	*n*	CU A+ (*n* = 63)	*n*	MCI A+ (*n* = 58)	*n*	Control CU A− T− (*n* = 81)	*n*	CU A+ T+ (*n* = 20)	n	MCI A+ T+ (*n* = 40)	*n*
Age (years), mean (SD)	69.0 (5.8)	100	71.4 (5.4)	63	72.4 (7.3)	58	68.7 (5.8)	81	73.5 (5.0)	20	72.0 (6.7)	40
Education (years), mean (SD)	16.8 (2.5)	100	16.5 (2.3)	63	16.4 (2.6)	58	16.6 (2.5)	81	16.8 (2.0)	20	16.5 (2.5)	40
Baseline MMSE, mean (SD)	29.1 (1.0)	98	28.8 (1.4)	63	27.4 (2.0)	58	29.1 (1.0)	80	28.4 (1.7)	20	27.1 (2.1)	40
Follow‐up (months)		100		63		58		81		20		40
Mean (SD)	35.4 (17.2)		27.5 (13.2)		26.0 (12.8)		35.9 (16.9)		29.9 (13.5)		23.3 (9.5)	
Median	34.2		24.4		24.2		39.4		24.6		23.8	
APOE ε4 carrier‐positive, *n* (%)	27 (27)	99	40 (65)	62	35 (67)	52	19 (24)	80	15 (75)	20	26 (74)	35
Female sex, *n* (%)	60 (60)	100	42 (67)	63	26 (45)	58	47 (58)	81	14 (70)	20	21 (52)	40
Baseline CDR score, *n* (%)		96		63		58		78		20		40
0	89 (93)		61 (97)		4 (7)		72 (92)		19 (95)		1 (2.5)	
0.5	7 (7)		2 (3)		52 (90)		6 (8)		1 (5)		38 (95)	
1	0		0		2 (3)		0		0		1 (2.5)	

MK‐6240 cohort (*N* = 168)	Control (CU A−) (*n* = 85)	*n*	CU A+ (*n* = 44)	*n*	MCI A+ (*n* = 24)	*n*	Control CU A− T− (*n* = 72)	*n*	CU A+ T+ (*n* = 21)	n	MCI A+ T+ (*n* = 17)	*n*
Age (years), mean (SD)	71.4 (8.0)	85	75.6 (7.1)	44	71.9 (7.0)	24	71.3 (8.4)	72	75.2 (6.1)	21	70.6 (7.2)	17
Education (years), mean (SD)	14.4 (3.0)	61	13.1 (2.8)	33	11.8 (2.5)	13	14.4 (3.1)	53	13.2 (2.5)	16	12.5 (2.2)	8
Baseline MMSE, mean (SD)	28.9 (1.2)	85	28.1 (1.6)	44	25.4 (3.5)	24	28.8 (1.2)	72	28.0 (1.8)	21	24.9 (3.8)	17
Follow‐up (months)		85		44		24		72		21		17
Mean (SD)	19.0 (5.2)		19.6 (6.4)		16.7 (6.6)		18.7 (5.2)		20.9 (6.5)		16.6 (7.3)	
Median	18.1		19.4		14.8		17.9		22.4		15.4	
APOE ε4 carrier‐positive, *n* (%)	14 (24)	59	19 (58)	33	7 (50)	14	12 (24)	51	10 (62)	16	5 (56)	9
Female sex, *n* (%)	38 (45)	85	25 (57)	44	16 (67)	24	29 (40)	72	15 (71)	21	10 (59)	17
Baseline CDR score, *n* (%)		79		39		21		67		20		15
0	75 (95)		31 (79)		3 (14)		63 (94)		16 (80)		2 (13)	
0.5	4 (5)		8 (21)		18 (86)		4 (6)		4 (20)		13 (87)	

Abbreviations: A+, amyloid‐positive; APOE ε4, apolipoprotein E epsilon 4; CDR, clinical dementia rating; CU, cognitively unimpaired; MCI, mild cognitive impairment; MMSE, Mini‐Mental State Examination; PET, positron emission tomography; SD, standard deviation; T+, tau PET positive.

### 2.2 Ethics

This study was conducted according to Good Clinical Practice guidelines in accordance with the ethical principles in the Declaration of Helsinki. Participant data were previously collected by their respective longitudinal cohort studies. Study participants gave written informed consent at the time of enrollment in their respective studies, which had been approved by each participating site's Institutional Review Board.

### 2.3 Image acquisition and processing

#### 2.3.1 Tau PET data acquisition

In the cohort scanned with the MK‐6240 tracer, we analyzed tau PET images acquired between 90 and 110 min in participants from AIBL and between 90 and 120 min in participants from Cerveau after intravenous bolus injection. In the Flortaucipir cohort, we analyzed tau PET images acquired between 75 and 105 min after intravenous bolus injection of ^18^F‐AV1451 tracer as per the standardized protocol for ADNI.^36^

#### 2.3.2 Tau PET data processing

A PET image processing pipeline assembled using components of the software packages AFNI,^41^ FreeSurfer,^42^ ANTs,^43^ and customized command line and R functions was used to process tau PET and MRI images to calculate voxel‐wise SUVRs using the cerebellar gray as the reference region (Figure S1).^44^, ^45^, ^46^ We only used data that overlapped with the 90‐ to 120‐min window for the MK‐6240 cohort and 75 to 105 min for the Flortaucipir cohort, using a linear weighting factor for each frame that reflects its overlap with the range. Images from dynamic tau PET scans were registered to minimize within scan motion. For participants with only cross‐sectional scans the average of the registered tau PET scan was used as reference to which T1 MRI scans were aligned. For participants with longitudinal tau PET scans, a between‐scan registration was also carried out between baseline (BL) and the first follow‐up (FU1), and images from both scans were aligned to the spatial midpoint between BL and FU1 visits. The average of midpoint registered BL scans was used as reference for MRI registration and subsequent FU tau PET scans. To minimize resampling‐induced smoothing bias, within‐ and across‐scan spatial transformations were combined and applied to the tau PET frames in one step. The T1 images were then co‐registered to the reference PET image. For the ADNI cohort, we equalized the smoothness of the noise across participants’ longitudinal tau PET scans using the approach developed by Iaccarino et al.,^38^ which assessed the smoothness (using AFNI's 3dFWHMx), then uniformly smoothed each participant's longitudinal PET data to the highest smoothness estimate (using 3dBlurToFWHM).^41^, ^47^, ^48^

#### 2.3.3 MRI data acquisition

Participants were imaged using a three‐dimensional (3D) T1‐weighted magnetization‐prepared rapid gradient echo (MPRAGE) sequence. For the AIBL cohort, the participants received an MRI scan using the ADNI 3D MPRAGE sequence, with 1 × 1 mm in‐plane resolution and 1.2 mm slice thickness. For Cerveau data, the T1 resolution was in the range of 0.8 isotropic to 1 mm isotropic resolution. Scans in the ADNI cohort were acquired with a standardized protocol across sites.^49^

#### 2.3.4 MR data analysis

For all participants, the baseline T1 MRI was resampled to an isotropic resolution of 1 mm^3^ and truncated along the inferior direction to remove excessive neck coverage from the images. The truncated T1 MRI was segmented using the FreeSurfer toolkit, which parcellates the brain into anatomical ROIs.

### 2.4 Measures of tau changes in the cortex

The two tau PET analysis methods designed to capture tau spread are detailed below. In addition, tau PET progression was quantified by computing SUVR change in anatomically defined ROIs as commonly carried out in the literature and in connectome‐based ROIs (Supplement methods; Figure S2).^50^

#### 2.4.1 Quantifying SPOT in the cortex

**FIGURE 1:**
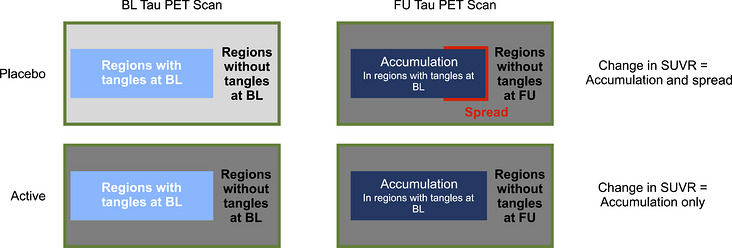
Schematic for spatial progression of tauopathy (SPOT). SPOT is a tau PET analysis method designed to measure tau spread. This schematic depicts cortical regions with NFTs (light blue) within the total cortical area (green border) at baseline. Over time, the cortical regions with NFTs at baseline can accumulate more tau (dark blue), and tau can spread to other regions that did not have NFTs at baseline (red). SPOT is calculated as the volume at follow‐up with NFTs (dark blue + red) minus the volume at baseline with NFTs (light blue). BL, baseline; FU, follow‐up; NFT, neurofibrillary tangle; PET, positron emission tomography; SUVR, standardized uptake value ratio.

The SPOT approach measures spread by computing the change in the fraction of cortex where voxels have elevated uptake. SPOT was calculated as the total volume of voxels exceeding the tracer‐specific SUVR threshold at the follow‐up visit minus the total volume of voxels exceeding the same SUVR threshold at the baseline visit (Figure 1). For each of the two tracers, the SUVR threshold was set as the median +2 median absolute deviation (MAD) of cortex‐wide SUVR observed in tracer‐matched CU A− cohorts. The resultant thresholds, applied uniformly across the brain, were similar for both tracers at SUVR of 1.2.

#### 2.4.2 Tau‐naïve ROI method: assessing SUVR changes across regions with no evidence of NFTs

**FIGURE 2:**
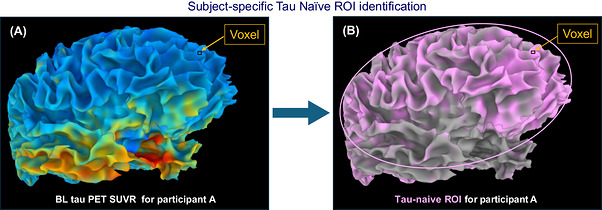
Tau‐naïve ROI method. In this method, SUVR changes are assessed across regions with no evidence of NFTs, as illustrated in this image of white matter surface created by FreeSurfer.^44^ (A) Voxel‐wise tau PET SUVR at baseline is projected on corresponding vertices on the white matter surface. The color at each vertex represents the amount of SUVR measured by tau PET: Higher levels of tau are represented by yellow and red, while lower levels of tau are represented by blue. (B) A voxel (black square) is marked as being tau‐naïve (pink) if its baseline tau PET SUVR is within 1 standard deviation of tau PET SUVR at that location in a control group of amyloid‐free cohort. PET, positron emission tomography; ROI, region of interest; SUVR, standardized uptake value ratio.

The tau‐naïve ROI method (Figure 2) defines spread as the change at a follow‐up time point in tau PET SUVR assessed over a region with no evidence of NFTs at baseline. This individual‐specific ROI was defined as an aggregate of all gray matter voxels within the median ± 1 MAD of the spatially corresponding median SUVR in the tracer‐matched control cohort (Figure S3). The use of the ± 1 MAD window for tau‐naïve ROI was deliberately conservative to minimize the inclusion of voxels with even slight elevations of NFTs as measured by PET. This narrow window was selected to maximize the likelihood of detecting a treatment effect for therapies that are hypothesized to interfere with tau spread, that is, stop the extracellular soluble tau from spreading to unaffected areas and initiating the de novo formation of intracellular NFTs in these previously unaffected areas. Maps of median and standard deviations in the control cohorts were computed over each of the 200 cortical ROIs and 32 subcortical ROIs defined using the Schaefer cortical atlas and Tian subcortical atlas.^51^, ^52^ The tiling by the 232 ROIs was used as a smoothing of the median and variation maps to reduce sensitivity to noise. While the normalization parameters were constant over each of the 232 ROIs, the resultant tau‐naïve ROI was generated at the PET scan resolution since the tau‐naïve assignment was carried out voxel by voxel.

**FIGURE 3:**
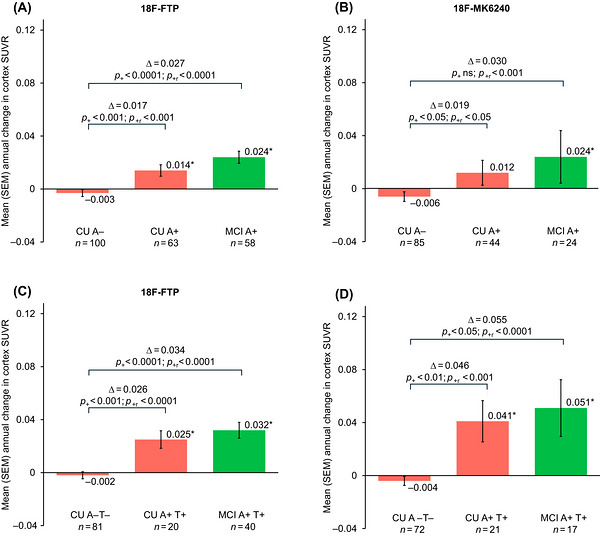
Annualized change in tau PET SUVR average over the cortex in amyloid‐positive participants and in controls in the Flortaucipir and MK‐6240 cohorts. Mean (SEM) annualized change in SUVR is demonstrated in the CU A+ and MCI A+ groups and the corresponding control CU A− group (A and B) and in the CU A+T+ and MCI A+T+ groups and the corresponding control CU A−T− group (C and D) in the Flortaucipir and MK‐6240 cohorts. ^*^ Significantly different from zero (*p* < 0.05 using a one‐sample *t*‐test). A+, amyloid‐positive; A−, amyloid‐negative; CU, cognitively unimpaired; MCI, mild cognitive impairment; *n*, number of participants; *p*+, one‐sided *p* value from parametric test for difference in means; *p*+*r*, one‐sided *p* value from non‐parametric (outlier robust) test; PET, positron emission tomography; SEM, standard error of the mean; SPOT, spatial progression of tauopathy; SUVR, standardized uptake value ratio; *T*+, tau PET positive.

**FIGURE 4:**
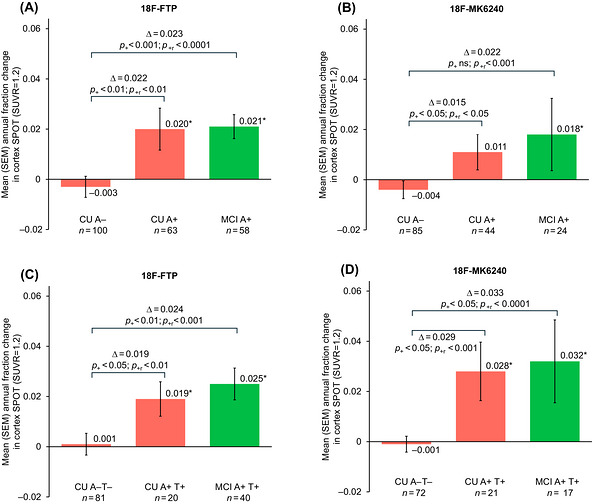
Spatial progression of tauopathy (SPOT) results in amyloid‐positive participants and in controls in the Flortaucipir and MK‐6240 cohorts. Mean (SEM) annualized changes in SPOT in CU A+ and MCI A+ groups and the corresponding control CU A− group (A and B) and in the CU A+T+ and MCI A+T+ groups and the corresponding control CU A−T− group (C and D) in the Flortaucipir and MK‐6240 cohorts. ^*^Significantly different from zero (*p* < 0.05 using a one‐sample *t*‐test). A+, amyloid‐positive; A−, amyloid‐negative; CI, confidence interval; CU, cognitively unimpaired; MCI, mild cognitive impairment; *n*, number of participants; *p*+, one‐sided *p* value from parametric test for difference in means; *p*+*r*, one‐sided *p* value from non‐parametric (outlier robust) test; PET, positron emission tomography; SEM, standard error of the mean; SPOT, spatial progression of tauopathy; SUVR, standardized uptake value ratio; T+, tau PET positive.

**FIGURE 5:**
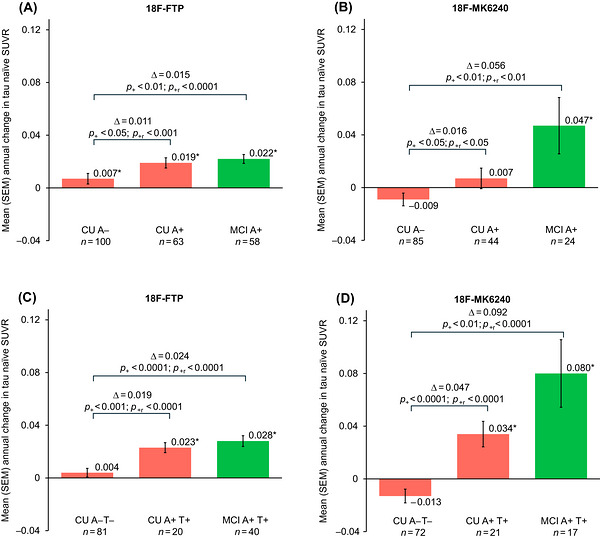
Tau‐naïve ROI results in amyloid‐positive participants versus controls in Flortaucipir and MK‐6240 cohorts. Mean (SEM) change in SUVR in the tau‐naïve ROI is demonstrated in CU A+ and MCI A+ groups and the corresponding control CU A− group (A and B) and in the CU A+T+ and MCI A+T+ groups and the corresponding control CU A−T− group (C and D) in the Flortaucipir and MK‐6240 cohorts. *Significantly different from zero (*p* < 0.05 using a one‐sample *t*‐test). A+, amyloid‐positive; A−, amyloid‐negative; CU, cognitively unimpaired; MCI, mild cognitive impairment; n, number of participants; p+, one‐sided *p* value from parametric test for difference in means; *p*+*r*, one‐sided *p* value from non‐parametric (outlier robust) test; PET, positron emission tomography; SEM, standard error of the mean; SPOT, spatial progression of tauopathy; SUVR, standardized uptake value ratio; *T*+, tau PET positive.

The reported delta is the difference in the mean values between cohorts under comparison. *P* values reported are one‐sided *p* values (alternative = “greater”) from a parametric *t*‐test (Welch's two sample *t*‐test) for the difference in means (indicated by *p*+ in Figures 3, 4, 5). The *p* value from the non‐parametric (outlier robust) test (Wilcoxon rank‐sum test) are also provided in the figures (indicated by *p*+*r* in Figures 3, 4, and 5). These associations among diagnostic groups were also tested using ordinary least squares regression, including age and sex as covariates.

## 3 RESULTS

### 3.1 Baseline characteristics of longitudinal cohorts

Longitudinal tau PET data were analyzed from a total of 221 participants in the Flortaucipir cohort and 168 participants in the MK‐6240 cohort. Baseline demographic and clinical characteristics of the participants who had at least two tau PET scans from the Flortaucipir or MK‐6240 cohorts are presented in the Table 1 study group, according to cognitive (CU or MCI), amyloid (A+ or A−), and tau (T+ or T−) status.

To create tracer‐specific normative distributions of tau PET SUVR, we analyzed scans of cognitively unimpaired amyloid negative (CU A−) participants that were available at the time of method development. The tracer‐specific control cohort consisted of 290 participants from the Flortaucipir cohort and 237 participants from the MK‐6240 cohort (Table S1).

### 3.2 Overview

SUVR change in the whole cortex was larger in the CU A+ and MCI A+ groups compared to the CU A− control group (Figure 3). SPOT and SUVR change in tau‐naïve ROI were larger in the CU A+ and MCI A+ groups compared to the CU A− group (Figures 4 and 5). Similar patterns were observed when the CU A+T+ and MCI A+T+ versus CU A−T− groups were compared (Figures 4 and 5). Changes in A+T+ groups were numerically larger than changes in their respective the A+ group in all of these tau PET measures, except for SPOT in the Flortaucipir cohort, where changes in the CU A+ group were comparable to changes in the CU A+T+ group. Differences were consistent across flortaucipir and MK‐6240 tracers (Figures 3, 4, 5). These results confirm that the SPOT and tau‐naïve ROI methods can detect tau progression in the presence of AD pathology. Results for the analysis of SUVR change in the whole cortex, SPOT method, and tau‐naïve ROI method are described individually below. Qualitatively similar results were obtained when repeating the comparisons with age and sex as covariates in the models to control for differences between the groups (Table S2).

### 3.3 Annualized SUVR change in whole cortex

Annualized SUVR change in the whole cortex is presented in Figure 3, comparing CU A+ and MCI A+ participants to CU A− controls in the Flortaucipir (Figure 3A) and MK‐6240 (Figure 3B) cohorts. The annualized change in SUVR was significantly higher in the CU A+ (Flortaucipir delta = 0.017, *p* < 0.001; MK‐6240 delta = 0.019, *p* < 0.05) and MCI A+ (Flortaucipir delta = 0.027, *p* < 0.0001) groups compared to the CU A− control group. A similar pattern was observed when comparing the tau‐positive subset of CU A+T+ (Flortaucipir delta = 0.026, *p* < 0.001; MK‐6240 delta = 0.046, *p* < 0.01) and MCI A+T+ (Flortaucipir delta = 0.034, *p* < 0.0001; MK‐6240 delta = 0.055, *p* < 0.05) groups to the CU A−T− control group (Figure 3C,D). These results from the cortex‐wide analysis of SUVR change serve as a reference for the subsequent results using the two tau spread analysis methods.

### 3.4 SPOT method

Using the SPOT analysis method, the annualized average SPOT was greater than zero in the CU A+ and MCI A+ groups using both flortaucipir and MK‐6240 tracers (Figure 4A,B). However, the annualized average SPOT did not reach significance (*p* > 0.05) for the CU A+ group with MK‐6240. The annualized average SPOT of the CU A+ (Flortaucipir delta = 0.22, *p* < 0.01; MK‐6240 delta = 0.015, *p* < 0.05) and MCI A+ (Flortaucipir delta = 0.023, *p* < 0.001) groups were significantly larger compared to the annualized average SPOT in the CU A− control group (Figure 4A,B). When analyzing the tau‐positive subsets of the CU A+T+ (Flortaucipir delta = 0.019, *p* < 0.05; MK‐6240 delta = 0.029, *p* < 0.05) and MCI A+T+ (Flortaucipir delta = 0.024, *p* < 0.01; MK‐6240 delta = 0.033, *p* < 0.05) groups compared to the CU A−T− control group, a similar pattern emerged but with a larger effect size than the comparison between the broader CU A+ and MCI A+ versus CU A− groups (Figure 4C,D). Overall, results obtained from SPOT were qualitatively similar to the results obtained for the cortex‐wide annualized change in SUVR.

### 3.5 Tau‐naïve ROI method

Using the tau‐naïve ROI analysis method, the annualized change in SUVR in the tau‐naïve ROI was greater than zero in the CU A+ and MCI A+ groups using both flortaucipir and MK‐6240 tracers (Figure 5A,B). However, the difference did not reach significance (*p* > 0.05) for the CU A+ group with MK‐6240. The annualized change in SUVR in the tau‐naïve ROI in the CU A+ (Flortaucipir delta = 0.011, *p* < 0.05; MK‐6240 delta = 0.016, *p* < 0.05) and MCI A+ (Flortaucipir delta = 0.015, *p* < 0.01; MK‐6240 delta = 0.056, *p* < 0.01) groups were significantly larger compared to the annualized change in SUVR in the CU A− control group (Figure 5A,B). Analysis of the tau‐positive subsets of the CU A+T+ (Flortaucipir delta = 0.019, *p* < 0.001; MK‐6240 delta = 0.047, *p* < 0.0001) and MCI A+T+ (Flortaucipir delta = 0.024, *p* < 0.0001; MK‐6240 delta = 0.092, *p* < 0.01) groups compared to the CU A−T− control group revealed the emergence of a similar pattern but with a larger effect size than that between the broader CU A+ and MCI A+ versus CU A− groups (Figure 5C,D).

### 3.6 Effect size of SPOT and SUVR change measures in amyloid‐ and tau‐positive participants

**FIGURE 6:**
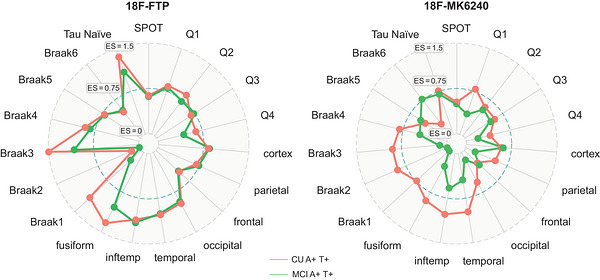
Effect size of SPOT and SUVR change measures in amyloid‐ and tau‐positive participants in Flortaucipir and MK‐6240 cohorts. Spider plots showing effect size of observed longitudinal change across SPOT, tau‐naïve ROI, connectome‐based ROIs (Q1, Q2, Q3, Q4), and anatomically defined ROIs for the Flortaucipir (A) and MK‐6240 (B) cohorts. A+, amyloid‐positive; CU, cognitively unimpaired; ES, effect size; inftemp, inferior temporal cortex; MCI, mild cognitive impairment; PET, positron emission tomography; Q, quadrant; ROI, region of interest; SUVR, standardized uptake value ratio; *T*+, tau PET positive.

The spider plots (Figure 6A,B) show the effect sizes of SPOT and SUVR change measures (calculated as annualized SUVR change/standard deviation) in the tau‐naïve, anatomically defined, and connectome‐based ROIs for CU A+T+ and MCI A+T+ participants in both tracer cohorts. In both tracer cohorts, SUVR changes in tau‐naïve ROI had an effect size of 0.75 or higher in both the CU A+T+ and MCI A+T+ groups. The SPOT methodology yielded effect sizes of approximately 0.6 in the Flortaucipir cohort and approximately 0.5 in the MK‐6240 cohort. While the effect sizes in SPOT and the tau‐naïve ROI were not the highest (e.g., note the high effect sizes observed in the inferior temporal lobe and Braak 3 regions), it compared favorably overall and showed consistent performance across both tracers and cohorts. The small sample sizes and large number of comparisons precluded statistical comparisons for which this study was not powered.

## 4 DISCUSSION

In this study, we presented SPOT and tau‐naïve ROI as two tau PET analysis methods designed to measure the spread of tau – detection of new NFTs appearing by tau PET in brain regions that showed no signal at baseline. These approaches differ from more traditional tau PET analysis methods that measure changes in tau in anatomically defined brain regions, regardless of the levels of NFTs at baseline. While traditional anatomically defined measures of SUVR are useful for evaluating tau accumulation, the SPOT and tau‐naïve ROI methods provide complementary information accentuating TSS. Using longitudinal observational data, we demonstrate that these two approaches can detect the tau progression expected in A+ participants, in a manner that reflects changes observed using traditional anatomically defined ROI‐based approaches. Results were qualitatively consistent across both tracer cohorts, suggesting that tau spread measures have comparable performance regardless of tracer type. In the setting of a natural history cohort, it is expected that the tau spread measures such as SPOT and tau‐naïve ROI methods will be similar to traditional measures of SUVR change in anatomically defined regions, as the results of this study have demonstrated. While spread measures may be advantageous in observational studies,^32^, ^53^ our focus here was on their potential for increased ability to detect treatment effects in disease modifying trials. Specifically, for treatments expected to impact tau spreading rather than continued local accumulation in regions with NFTs at baseline, tau spread measures may better detect a treatment effect. For this reason, they serve as complementary measures to traditional regional SUVR to yield a more comprehensive evaluation of tau progression. In principle, tau spread measures may be more sensitive to treatment effects of drugs that target tau spread, although this will need to be confirmed in the setting of an interventional clinical trial.

Previously published methods on tau spread measures using tau PET are similar to the SPOT method.^28^ Maass et al. quantified whole‐brain measure of tau spread using count of voxels above a certain SUVR threshold.^29^ In a longitudinal tau PET imaging study by Pontecorvo et al., global cortical flortaucipir retention was summarized by a SUVR for each participant, with target volumes of interest consisting of weighted average of voxels.^24^ Doering et al. defined TSS as the fraction of the cortex where tau has spread (as defined by a *Z*‐score of > 1.96, as derived by a voxel‐wise Z‐scoring approach).^31^ Gerard et al. defined the spatial extent of tauopathy (EOT) as the percentage of voxels with SUVR ≥ 1.3.^32^ Similarly, the SPOT method presented in this study required selection of a SUVR threshold value of 1.2 to determine tauopathy. This value was selected to be high enough to yield differences between diagnostic categories, but low enough to reflect an early stage of disease. We note that with the exception of using a fixed threshold across the brain, this approach is comparable to taking the difference between spatial extent of tau measures,^31^, ^32^ scaled by whole cortex volume. Spatial extent was also found by Coomans et al. to improve concordance with visual reads for tau PET positivity.^53^

The second proposed approach for the assessment of tau spread is the novel tau‐naïve ROI method, which measures changes in tau PET in regions that previously had no evidence of NFTs. Like the SPOT method, the tau‐naïve ROI method is designed to reduce the dilution of spread‐targeting treatment effects should the intervention fail to slow the continued accumulation of NFTs in brain regions where they are already established. The tau‐naïve ROI is an individual‐specific aggregate of NFT‐free regions at baseline, taking into account an AD patient's unique topography of tau pathology. As such, the tau‐naïve ROI may be able capture changes in tau pathology in AD patients with atypical tau patterns, which may not be captured with standard anatomical ROIs. We demonstrated in observational cohorts that SUVR changes over the tau‐naïve ROI have an effect size comparable to that of other ROIs, making the detection of a treatment effect on spread to the tau‐naïve brain possible. This effect size was numerically higher when analyzing the subgroup of participants who were also tau positive at baseline. We note, however, that the present study does not allow for the comparison of effect sizes across tracers because of cohort differences. However, a recent study comparing flortaucipir to MK‐6240 in matched cohorts found the latter to be more sensitive at detecting change in preclinical AD.^54^

There are several limitations to the methods presented in this study. Brain volume decreases over time in the elderly population are exacerbated in neurodegenerative diseases.^55^ For this reason, it is imperative to consider effects on tau PET in concert with effects on neurodegeneration. Partial volume correction methods may serve to reduce the bias, though possibly at the expense of increased variance.^56^ Such gray matter volume decreases may bias tau PET measures as follows. If we approximate the PET signal in a voxel as the product of gray matter volume (V_g_) by NFT concentration (C_NFT_), a reduction in V_g_ while C_NFT_ is increasing would bias tau PET change measures lower than they would have been if V_g_ were constant. This bias may be diminished in the active treatment arm if neurodegeneration is halted, making the contrast with the placebo arm less pronounced than it should be. While the effect of atrophy may bias SPOT and SUVR change over tau‐naïve regions, the bias is expected to be comparable for SPOT to that observed for SUVR measures from traditional anatomically defined regions. For SUVR change over tau‐naïve regions, the bias should be lower than for the other approaches because the SUVR change is derived over regions least affected by NFTs at baseline and therefore least likely to exhibit NFT‐induced neurodegeneration.

Another potential source of bias is partial volume and point spread function effects, though these are unlikely to have a measurable impact when assessing treatment effects on spread in clinical trials as they impact all treatment arms equally.

While both SPOT and tau‐naïve ROI are designed to capture tau spread, they do so differently. A comparison of their ability to detect a treatment effect will need to be determined empirically using clinical trial data. These methods for measuring tau spread may be useful in very early stages of the AD disease spectrum but not in later stages of AD due to a saturation effect when tau is widespread. For this reason, our analyses focused on CU and MCI participants, but not on patients with later‐stage AD.

In conclusion, this study presented two tau PET analysis methods designed to detect tau spatial spread in AD. Overall, a measurable effect size was found using SPOT and the tau‐naïve ROI consistently across tau PET tracers and cognitive status groups, indicating that the SPOT and tau‐naïve ROI are viable methods and potential endpoints in trials evaluating treatments targeting tau spread. Quantitative measures of tau spread and accumulation play a critical role in clinical trials in AD, especially in preclinical or early AD, as pharmacodynamic biomarkers and potentially surrogate biomarkers for clinical progression in AD. The tau PET analysis methods presented in this study are designed specifically to measure tau spread and so will serve as useful complementary tools in clinical trials evaluating therapies that may target or impact tau spread, as will investigational drugs with other mechanisms of action that may have a downstream impact on tau spread. Thus, SPOT and tau‐naïve ROI analysis methods should be considered alongside traditional anatomically based measures of SUVR change to provide a more complete assessment of treatment effects on tau pathology.

## AUTHOR CONTRIBUTIONS

**Janice Wong**: Investigation; writing–original draft; writing–review and editing; visualization. **Tina Wang**: Software; validation; formal analysis; writing–review and editing; visualization. **Ritobrato Datta**: Software; validation; formal analysis; writing–review and editing; visualization; **Rouhollah O. Abdollahi**: Software; validation; formal analysis; writing–review and editing. **Jingwei Li**: Software; validation; formal analysis; writing–review and editing. **Christopher C Rowe**: Investigation; writing–review and editing. **David Henley**: Investigation; writing–review and editing; visualization. **Hartmuth C. Kolb**: Conceptualization; methodology; investigation; writing–review and editing. **Ziad S. Saad**: Conceptualization; methodology; software; validation; formal analysis; investigation; writing–review and editing; visualization; supervision. **Alzheimer's Disease Neuroimaging Initiative**: Data curation; writing–review and editing.

## CONSENT STATEMENT

This non‐interventional study was conducted using third‐party anonymized participant cohorts, and participant consent for the use of the anonymized third‐party data for this study was therefore not required.

## CONFLICT OF INTEREST STATEMENT

All authors are current or past employees of Johnson & Johnson and may hold company stock. Author disclosures are available in the Supporting Information.

## Supporting information




**Supporting Information**: alz71573‐sup‐0001‐ICMJE.pdf

**Supporting Information**: alz71573‐sup‐0002‐SupMat.docx

## References

[1] Braak H , Braak E . Neuropathological stageing of Alzheimer‐related changes. Acta Neuropathol. 1991;82:239‐259. 1759558 10.1007/BF00308809

[2] Thal DR , Attems J , Ewers M . Spreading of amyloid, tau, and microvascular pathology in Alzheimer's disease: findings from neuropathological and neuroimaging studies. J Alzheimers Dis. 2014;42(Suppl 4):S421‐S429. 25227313 10.3233/JAD-141461

[3] Amorim IS , Challal S , Cistarelli L , et al. A seeding‐based neuronal model of tau aggregation for use in drug discovery. PLoS One. 2023;18:e0283941. 37014877 10.1371/journal.pone.0283941 PMC10072482

[4] Medeiros R , Baglietto‐Vargas D , LaFerla FM . The role of tau in Alzheimer's disease and related disorders. CNS Neurosci Ther. 2011;17:514‐524. 20553310 10.1111/j.1755-5949.2010.00177.x PMC4072215

[5] Walker LC . Prion‐like mechanisms in Alzheimer disease. Handb Clin Neurol. 2018;153:303‐319. 29887142 10.1016/B978-0-444-63945-5.00016-7 PMC6375694

[6] Meisl G , Hidari E , Allinson K , Rittman T , et al. In vivo rate‐determining steps of tau seed accumulation in Alzheimer's disease. Sci Adv. 2021;7:eabh1448. 34714685 10.1126/sciadv.abh1448 PMC8555892

[7] Mohamed NV , Herrou T , Plouffe V , Piperno N , Leclerc N . Spreading of tau pathology in Alzheimer's disease by cell‐to‐cell transmission. Eur J Neurosci. 2013;37:1939‐1948. 23773063 10.1111/ejn.12229

[8] Colom‐Cadena M , Davies C , Sirisi S , et al. Synaptic oligomeric tau in Alzheimer's disease—A potential culprit in the spread of tau pathology through the brain. Neuron. 2023;111:2170‐2183 e6. 37192625 10.1016/j.neuron.2023.04.020

[9] Schoonhoven DN , Coomans EM , Millan AP , et al. Tau protein spreads through functionally connected neurons in Alzheimer's disease: a combined MEG/PET study. Brain. 2023;146:4040‐4054. 37279597 10.1093/brain/awad189 PMC10545627

[10] Hoenig MC , Bischof GN , Seemiller J , et al. Networks of tau distribution in Alzheimer's disease. Brain. 2018;141:568‐581. 29315361 10.1093/brain/awx353

[11] Franzmeier N , Rubinski A , Neitzel J , et al. Functional connectivity associated with tau levels in ageing, Alzheimer's, and small vessel disease. Brain. 2019;142:1093‐1107. 30770704 10.1093/brain/awz026 PMC6439332

[12] Vogel JW , Iturria‐Medina Y , Strandberg OT , et al. Spread of pathological tau proteins through communicating neurons in human Alzheimer's disease. Nat Commun. 2020;11:2612. 32457389 10.1038/s41467-020-15701-2 PMC7251068

[13] Lee WJ , Brown JA , Kim HR , et al. Regional Abeta‐tau interactions promote onset and acceleration of Alzheimer's disease tau spreading. Neuron. 2022;110:1932‐1943 e5. 35443153 10.1016/j.neuron.2022.03.034 PMC9233123

[14] Budd Haeberlein S , Aisen PS , Barkhof F , et al. Two randomized phase 3 Studies of Aducanumab in early Alzheimer's disease. J Prev Alzheimers Dis. 2022;9:197‐210. 35542991 10.14283/jpad.2022.30

[15] van Dyck CH , Swanson CJ , Aisen P , et al. Lecanemab in early Alzheimer's disease. N Engl J Med. 2023;388:9‐21. 36449413 10.1056/NEJMoa2212948

[16] Sims JR , Zimmer JA , Evans CD , et al. Donanemab in early symptomatic Alzheimer disease: the TRAILBLAZER‐ALZ 2 randomized clinical trial. JAMA. 2023;330:512‐527. 37459141 10.1001/jama.2023.13239 PMC10352931

[17] Bierer LM , Hof PR , Purohit DP , et al. Neocortical neurofibrillary tangles correlate with dementia severity in Alzheimer's disease. Arch Neurol. 1995;52:81‐88. 7826280 10.1001/archneur.1995.00540250089017

[18] Nelson PT , Alafuzoff I , Bigio EH , et al. Correlation of Alzheimer disease neuropathologic changes with cognitive status: a review of the literature. J Neuropathol Exp Neurol. 2012;71:362‐381. 22487856 10.1097/NEN.0b013e31825018f7 PMC3560290

[19] Cho H , Choi JY , Hwang MS , et al. Tau PET in Alzheimer disease and mild cognitive impairment. Neurology. 2016;87:375‐383. 27358341 10.1212/WNL.0000000000002892

[20] Johnson KA , Schultz A , Betensky RA , et al. Tau positron emission tomographic imaging in aging and early Alzheimer disease. Ann Neurol. 2016;79:110‐119. 26505746 10.1002/ana.24546 PMC4738026

[21] Therriault J , Pascoal TA , Lussier FZ , et al. Biomarker modeling of Alzheimer's disease using PET‐based Braak staging. Nat Aging. 2022;2:526‐535. 37118445 10.1038/s43587-022-00204-0 PMC10154209

[22] Pontecorvo MJ , Devous MD, Sr , Navitsky M , et al. Relationships between flortaucipir PET tau binding and amyloid burden, clinical diagnosis, age and cognition. Brain. 2017;140:748‐763. 28077397 10.1093/brain/aww334 PMC5382945

[23] Ossenkoppele R , Smith R , Mattsson‐Carlgren N et al. Accuracy of Tau positron emission tomography as a prognostic marker in preclinical and prodromal alzheimer disease: a head‐to‐head comparison against amyloid positron emission tomography and magnetic resonance imaging. JAMA Neurol. 2021;78:961‐971. 34180956 10.1001/jamaneurol.2021.1858 PMC8240013

[24] Pontecorvo MJ , Devous MD , Kennedy I , et al. A multicentre longitudinal study of flortaucipir (18F) in normal ageing, mild cognitive impairment and Alzheimer's disease dementia. Brain. 2019;142:1723‐1735. 31009046 10.1093/brain/awz090 PMC6536847

[25] Bejanin A , Schonhaut DR , La Joie R , et al. Tau pathology and neurodegeneration contribute to cognitive impairment in Alzheimer's disease. Brain. 2017;140:3286‐3300. 29053874 10.1093/brain/awx243 PMC5841139

[26] Tanner JA , Rabinovici GD . Relationship between Tau and cognition in the evolution of alzheimer's disease: new insights from Tau PET. J Nucl Med. 2021;62:612‐613. 33277390 10.2967/jnumed.120.257824 PMC9364866

[27] Congdon EE , Ji C , Tetlow AM , Jiang Y , Sigurdsson EM . Tau‐targeting therapies for Alzheimer disease: current status and future directions. Nat Rev Neurol. 2023;19:715‐736. 37875627 10.1038/s41582-023-00883-2 PMC10965012

[28] Bollack A , Pemberton HG , Collij LE , et al. Longitudinal amyloid and tau PET imaging in Alzheimer's disease: a systematic review of methodologies and factors affecting quantification. Alzheimers Dement. 2023;19:5232‐5252. 37303269 10.1002/alz.13158

[29] Maass A , Landau S , Baker SL , et al. Comparison of multiple tau‐PET measures as biomarkers in aging and Alzheimer's disease. Neuroimage. 2017;157:448‐463. 28587897 10.1016/j.neuroimage.2017.05.058 PMC5814575

[30] Xu X , Ruan W , Liu F , et al. (18)F‐APN‐1607 Tau positron emission tomography imaging for evaluating disease progression in Alzheimer's disease. Front Aging Neurosci. 2021;13:789054. 35221982 10.3389/fnagi.2021.789054 PMC8868571

[31] Doering S , McCullough A , Gordon BA , et al. Deconstructing pathological tau by biological process in early stages of Alzheimer disease: a method for quantifying tau spatial spread in neuroimaging. EBioMedicine. 2024;103:105080. 38552342 10.1016/j.ebiom.2024.105080 PMC10995809

[32] Gerard T , Colmant L , Malotaux V et al. The spatial extent of tauopathy on [(18)F]MK‐6240 tau PET shows stronger association with cognitive performances than the standard uptake value ratio in Alzheimer's disease. Eur J Nucl Med Mol Imaging. 2024;51:1662‐1674. 38228971 10.1007/s00259-024-06603-2 PMC11043108

[33] Chien DT , Bahri S , Szardenings AK , et al. Early clinical PET imaging results with the novel PHF‐tau radioligand [F‐18]‐T807. J Alzheimers Dis. 2013;34:457‐468. 23234879 10.3233/JAD-122059

[34] Folstein MF , Folstein SE , McHugh PR . “Mini‐mental state”. A practical method for grading the cognitive state of patients for the clinician. J Psychiatr Res. 1975;12:189‐198. 1202204 10.1016/0022-3956(75)90026-6

[35] Krishnadas N , Dore V , Robertson JS , et al. Rates of regional tau accumulation in ageing and across the Alzheimer's disease continuum: an AIBL (18)F‐MK6240 PET study. EBioMedicine. 2023;88:104450. 36709581 10.1016/j.ebiom.2023.104450 PMC9900352

[36] ADNI ADNI study protocol. Accessed November 2024. 2024. https://adni.loni.usc.edu/methods/pet‐analysis‐method/pet‐analysis

[37] Winblad B , Palmer K , Kivipelto M , et al. Mild cognitive impairment–beyond controversies, towards a consensus: report of the international working group on mild cognitive impairment. J Intern Med. 2004;256:240‐246. 15324367 10.1111/j.1365-2796.2004.01380.x

[38] Petersen RC , Smith GE , Waring SC , Ivnik RJ , Tangalos EG , Kokmen E . Mild cognitive impairment: clinical characterization and outcome. Arch Neurol. 1999;56:303‐308. 10190820 10.1001/archneur.56.3.303

[39] Fowler C , Rainey‐Smith SR , Bird S , et al. Fifteen years of the Australian Imaging, Biomarkers and Lifestyle (AIBL) study: progress and observations from 2,359 older adults spanning the spectrum from cognitive normality to Alzheimer's disease. J Alzheimers Dis Rep. 2021;5:443‐468. 34368630 10.3233/ADR-210005 PMC8293663

[40] Ellis KA , Bush AI , Darby D , et al. The Australian Imaging, Biomarkers and Lifestyle (AIBL) study of aging: methodology and baseline characteristics of 1112 individuals recruited for a longitudinal study of Alzheimer's disease. Int Psychogeriatr. 2009;21:672‐687. 19470201 10.1017/S1041610209009405

[41] Cox RW . AFNI: software for analysis and visualization of functional magnetic resonance neuroimages. Comput Biomed Res. 1996;29:162‐173. 8812068 10.1006/cbmr.1996.0014

[42] Fischl B . FreeSurfer. Neuroimage. 2012;62:774‐781. 22248573 10.1016/j.neuroimage.2012.01.021 PMC3685476

[43] Avants BB , Tustison NJ , Song G , Cook PA , Klein A , Gee JC . A reproducible evaluation of ANTs similarity metric performance in brain image registration. Neuroimage. 2011;54:2033‐2044. 20851191 10.1016/j.neuroimage.2010.09.025 PMC3065962

[44] FreeSurfer. Accessed November 2024. https://surfer.nmr.mgh.harvard.edu

[45] Analysis of Functional NeuroImages (AFNI). Available at: Accessed November 2024. https://afni.nimh.nih.gov

[46] Advanced Normalization Tools (ANTS). Available at: Accessed November 2024. https://picsl.upenn.edu/software/ants/

[47] Cox RW , Chen G , Glen DR , Reynolds RC , Taylor PA . FMRI clustering in AFNI: false‐positive rates redux. Brain Connect. 2017;7:152‐171. 28398812 10.1089/brain.2016.0475 PMC5399747

[48] Iaccarino L , La Joie R , Koeppe R , et al. rPOP: robust PET‐only processing of community acquired heterogeneous amyloid‐PET data. Neuroimage. 2022;246:118775. 34890793 10.1016/j.neuroimage.2021.118775

[49] Jack CR, Jr , Bernstein MA , Borowski BJ , et al. Update on the magnetic resonance imaging core of the Alzheimer's disease neuroimaging initiative. Alzheimers Dement. 2010;6:212‐220. 20451869 10.1016/j.jalz.2010.03.004 PMC2886577

[50] Franzmeier N , Neitzel J , Rubinski A , et al. Functional brain architecture is associated with the rate of tau accumulation in Alzheimer's disease. Nat Commun. 2020;11:347. 31953405 10.1038/s41467-019-14159-1 PMC6969065

[51] Schaefer A , Kong R , Gordon EM , et al. Local‐global parcellation of the human cerebral cortex from intrinsic functional connectivity MRI. Cereb Cortex. 2018;28:3095‐3114. 28981612 10.1093/cercor/bhx179 PMC6095216

[52] Tian Y , Margulies DS , Breakspear M , Zalesky A . Topographic organization of the human subcortex unveiled with functional connectivity gradients. Nat Neurosci. 2020;23:1421‐1432. 32989295 10.1038/s41593-020-00711-6

[53] Coomans EM , van Tol B , Groot C , et al. Quantitation of PET spatial extent as a potential adjunct to visual interpretation of [(18)F]flortaucipir imaging: tAU‐SPEX. Eur J Nucl Med Mol Imaging. 2025;52:5135‐5149. 40481862 10.1007/s00259-025-07384-y PMC12589304

[54] Bourgeat P , Krishnadas N , Dore V , et al. Cross‐sectional and longitudinal comparison of tau imaging with 18f‐mk6240 and 18f‐flortaucipir in populations matched for age, mmse and brain beta‐amyloid burden. J Prev Alzheimers Dis. 2023;10:251‐258. 36946452 10.14283/jpad.2023.17

[55] Edmonds EC , Weigand AJ , Hatton SN , et al. Patterns of longitudinal cortical atrophy over 3 years in empirically derived MCI subtypes. Neurology. 2020;94:e2532‐e44. 32393648 10.1212/WNL.0000000000009462 PMC7455336

[56] Sanabria Bohorquez SM , Baker S , Manser PT , et al. Evaluation of partial volume correction and analysis of longitudinal [(18)F]GTP1 tau PET imaging in Alzheimer's disease using linear mixed‐effects models. Front Neuroimaging. 2024;3:1355402. 38606196 10.3389/fnimg.2024.1355402 PMC11008283

