# Supplementary material for: Tau positron emission tomography analysis methods for the quantification of tau spread in preclinical and early Alzheimer's disease
Source: Alzheimers Dement. 2026 Jun 23;22(6):e71573. doi: 10.1002/alz.71573 (PMC13290639; doi:10.1002/alz.71573)
Supplement: Supplementary file 2 — Supporting Information: alz71573‐sup‐0002‐SupMat.docx [file ALZ-22-e71573-s002.docx]

# SUPPLEMENT

# SUPPLEMENT METHODS

## Alignment of multiple 3D frames within a single PET imaging session

Within a session, four to six 3D tau PET images (5 min each) were acquired from 75 to 105 minutes for ADNI and 90 to 120 minutes for AIBL/Cerveau data. The 3D images were concatenated into a 4D image (3D+time). For each 4D PET image, a reference 3D volume was generated by averaging the 3D images across time. The individual 3D images were then aligned to this reference 3D image and head-motion parameters (volume-to-reference transform matrices and corresponding rotation and translation parameters) were estimated with 3dvolreg.

## Longitudinal alignment of PET images

When available, PET images were available from multiple sessions over time from the same participant, the pipeline detected and registered the images from baseline and follow-up sessions. Reference 3D PET images from baseline and follow-up sessions were used to generate a 3D template image that was unbiased with respect to any single time point. The reference 3D image from baseline was realigned to this unbiased template and all 4D images from baseline and follow-up were registered to the realigned baseline 3D PET image. Aligning longitudinal PET images from multiple sessions to the unbiased template space avoided large displacements that may occur if follow-up images are aligned to the baseline images. This approach minimized smoothing bias and reduced the random variation in the processing procedure and improved the robustness and sensitivity of the overall longitudinal analysis to detect small or subtle changes [1, 2].

## T1 to PET co-registration

Non-uniformity corrected T1 MR-images were co-registered to the realigned baseline 3D image with a six-parameter rigid registration by means of 3dAllineate using seven different cost functions. A visual QC process was used to select the best alignment or modify parameters to guide the alignment of T1 to PET, when needed.

## Normalization to MNI template

The co-registered T1 image was normalized to the MNI template (available in AFNI) using affine transformation with 12 degrees of freedom. For group level analyses, the voxelwise SUV and SUVR images were normalized to the MNI template using the transformation matrix obtained in the previous step. ROIs in the Schaefer and Tian atlases in MNI space were mapped to the native space using the inverse transformations of the matrix obtained in the T1 to MNI normalization step [3, 4].

## Regions of interest

ROIs were required in the different processing steps in the tau PET pipeline. As mentioned in the MRI processing section, anatomical regions of interest were identified using the T1 image. The FreeSurfer ROIs were re-labeled and combined using SUMA to create custom atlases (such as Braak regions). All ROIs were resampled and aligned to the PET images. For tau PET analysis, multiple ROIs (cerebellar gray, pons, whole cerebellum) were generated to be used as reference regions to compute SUVR maps.

## SUVR quantification

Participant-specific reference region (cerebellar gray) was chosen for computation of SUVR in the participant’s native space. The choice of reference region was determined from the literature and was an anatomical region devoid of specific tracer binding. Average SUVRs were calculated from different brain areas as defined by the ROIs for further statistical analysis.

## Results report

The ROIs defined in “MR Image Processing” section were used to calculate regional mean values from the output images after co-registration. Additionally, if cortical surface meshes were provided by the user, the output images could be interpolated on these meshes and be used to derive surface-based parameter estimates. Regional mean parameter values were saved in wide format ‘.csv’ files. This standardized data format simplified the subsequent analysis with statistical software, such as R.

### Quality control and visualization:

The tau PET pipeline includes both visual and automated quality control. Visual quality control (QC) was facilitated by the incorporation of a 3D/4D brain volume viewer in AFNI. This made it possible to visualize the output images of the longitudinal PET and T1 to PET co-registration and overlay of the ROIs onto the PET image without the need for additional software.

Visual QC was performed by viewing the co-registered PET to PET and T1 to PET images by overlaying one on top of the other and toggling between the two images. The visual QC was invoked once the data had been run through the automated pipeline and the outputs of all the seven cost functions had been computed. Visual inspection remained the gold-standard method for verifying the accuracy of PET-to-PET and T1-to-PET co-registration [5-9].

## Follow-up PET to baseline PET QC

The QC was done to ensure that the follow-up PET images were aligned to the baseline PET image. The QC was visual and leveraged the knowledge and experience of the adjudicator. The adjudicator toggled between the follow-up images registered to the baseline images and baseline PET images themselves and checked how well the images are aligned. If the alignment was acceptable (as defined by having no major head movement, and alignment of nose, ventricles, occipito-cerebellar junction), the adjudicator marked their impression onto a parameter log file that intakes the input from the adjudicator and applies the options to the follow-up image. If on the other hand, the alignment was not acceptable and needed to be fixed, the adjudicator attempted to make changes to the orientation of the follow-up image to match up with the baseline image. This was done in several ways by introducing rotations and translations on the follow-up image, changing the cost function that is needed to register the follow-up image to the baseline image by minimizing the high signal of the PET image, or clipping the follow-up or baseline image to remove the neck region from the image.

## High performance computing

The tau PET pipeline was optimized for high performance computing. The tau PET pipeline was distributed in a Docker container that contained all the software necessary to run the tau PET pipeline on any computing platform supporting such containers (i.e., where Docker or Singularity has been installed). The tau PET pipeline could therefore be run identically across a wide variety of computing environments. This not only facilitated the reproducibility of results but also allowed the tau PET pipeline to be deployed simultaneously across multiple computing nodes to analyze participants in parallel.

The ability to process large data sets in an easy, fast, and reproducible manner was essential, particularly in cases where parameters for a given algorithm needed to be optimized or where the performance of different algorithms at a given processing stage was being compared.

### Assessing tau spread in functionally connected regions

Prior studies provide converging evidence of a positive association between brain connectivity and the accumulation of tau pathology. The degree to which each brain region is connected to other brain regions (200 cortical regions and 32 subcortical regions) was derived from resting state functional MRI data from 500 healthy patients available from the Human Connectome Project using an in-house implementation of the analysis pipeline (Supplement figure 2) [3, 4, 10]. NFT levels were quantified by extracting the mean tau PET SUVR values for the 232 regions. For each patient, the NFT epicenter consisted of regions with the top 10% of NFT levels that were at least one SD above uptake in CU A- controls. All remaining regions were assigned a rank based on the strength of their average functional connectivity to the epicenter. Rank of connectivity to the epicenter was used to group regions into four quartiles (Q1 to Q4), with Q1 having the strongest connectivity to the epicenter.

# REFERENCES

[1] Reuter M, Fischl B. Avoiding asymmetry-induced bias in longitudinal image processing. Neuroimage. 2011;57:19-21.

[2] Reuter M, Schmansky NJ, Rosas HD, Fischl B. Within-subject template estimation for unbiased longitudinal image analysis. Neuroimage. 2012;61:1402-18.

[3] Schaefer A, Kong R, Gordon EM, Laumann TO, Zuo XN, Holmes AJ, et al. Local-Global Parcellation of the Human Cerebral Cortex from Intrinsic Functional Connectivity MRI. Cereb Cortex. 2018;28:3095-114.

[4] Tian Y, Margulies DS, Breakspear M, Zalesky A. Topographic organization of the human subcortex unveiled with functional connectivity gradients. Nat Neurosci. 2020;23:1421-32.

[5] Ge Y, Fitzpatrick JM, Votaw JR, Gadamsetty S, Maciunas RJ, Kessler RM, et al. Retrospective registration of PET and MR brain images: an algorithm and its stereotactic validation. J Comput Assist Tomogr. 1994;18:800-10.

[6] Andersson JL, Sundin A, Valind S. A method for coregistration of PET and MR brain images. J Nucl Med. 1995;36:1307-15.

[7] Alpert NM, Berdichevsky D, Levin Z, Morris ED, Fischman AJ. Improved methods for image registration. Neuroimage. 1996;3:10-8.

[8] Mutic S, Dempsey JF, Bosch WR, Low DA, Drzymala RE, Chao KS, et al. Multimodality image registration quality assurance for conformal three-dimensional treatment planning. Int J Radiat Oncol Biol Phys. 2001;51:255-60.

[9] DeLorenzo C, Klein A, Mikhno A, Gray N, Zanderigo F, Mann JJ, et al. A new method for assessing PET-MRI coregistration: SPIE; 2009.

[10] Franzmeier N, Neitzel J, Rubinski A, Smith R, Strandberg O, Ossenkoppele R, et al. Functional brain architecture is associated with the rate of tau accumulation in Alzheimer's disease. Nat Commun. 2020;11:347.

#
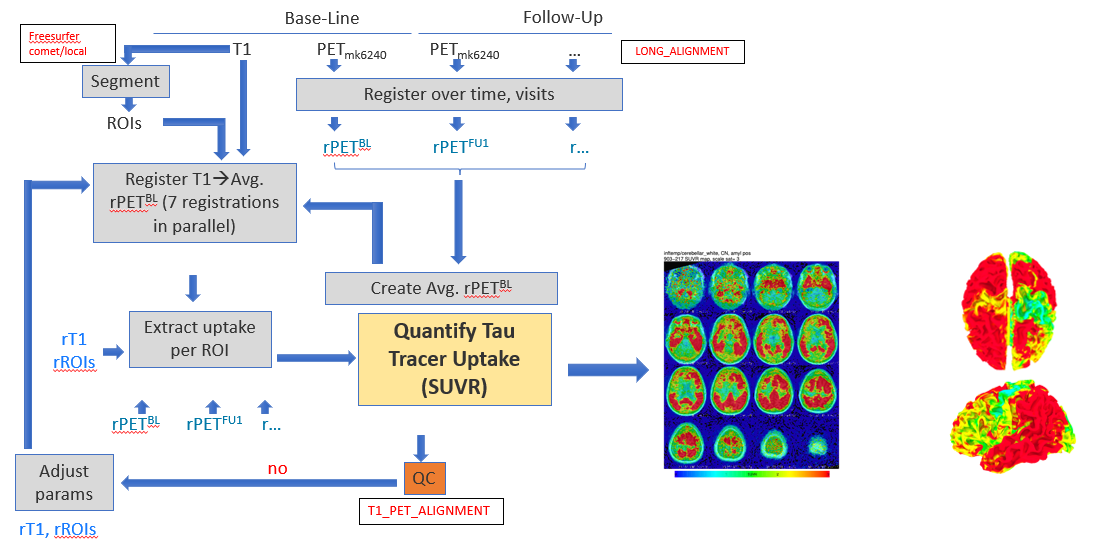
SUPPLEMENT FIGURES AND TABLES

## Supplement figure 1. Tau PET pipeline to quantify tau uptake in baseline (BL) and follow-up (FU) scans

Avg., average; BL, baseline; FU1, follow-up 1; PET, positron emission tomography; QC, quality control; ROI, region of interest; rPET, registered PET; SUVR, standardized uptake value ratio; T1, T1-weighted.


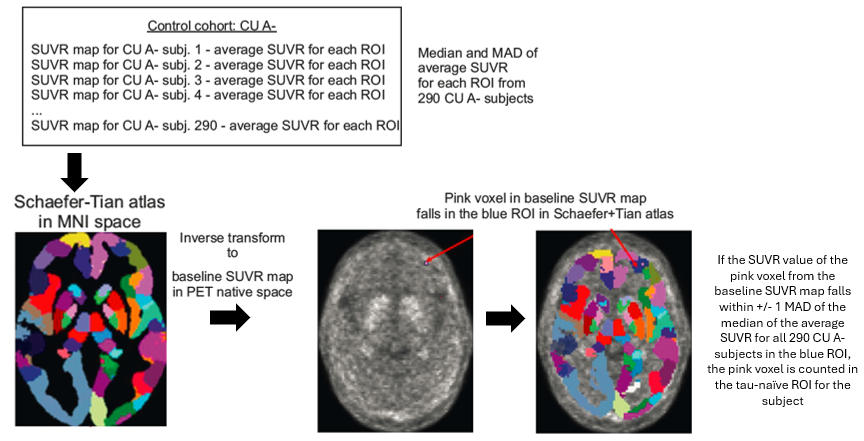


## Supplement figure 2. Predicting regions of strongest NFT increase using a functional brain connectivity approach based on Franzmeier et al. 2020[10]

BL, baseline; CU, cognitively unimpaired; FMRI, functional magnetic resonance imaging; FU, follow-up; HCP, Human Connectome Project; NFT, neurofibrillary tangle; PET, positron emission tomography; SD, standard deviation.


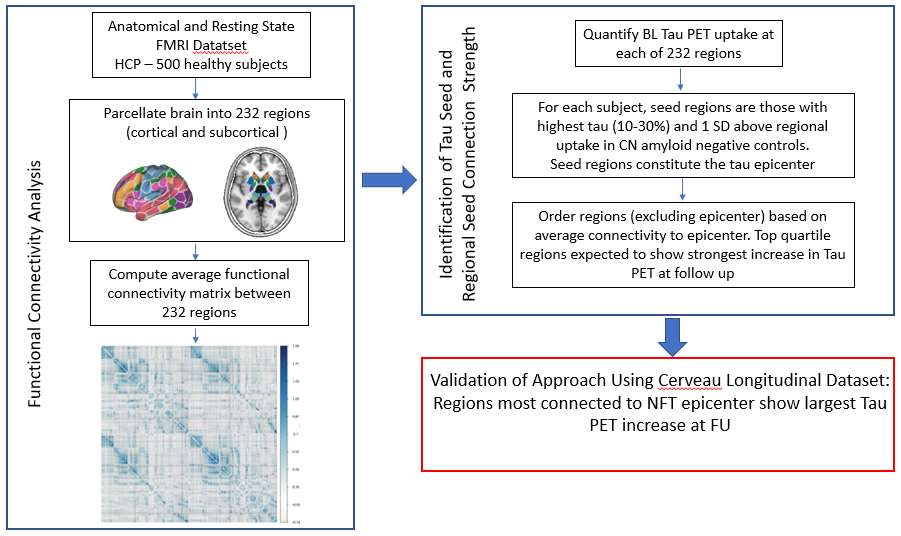


## Supplement figure 3. Tau-naïve ROI mapping using the Schaefer-Tian brain region atlas

CU A-, cognitively unimpaired amyloid-negative; MAD, Mean absolute deviation; PET, positron emission tomography; ROI, region of interest; Sub, participant; SUVR, standardized uptake value ratio.

## Supplement table 1: Baseline demographic and clinical characteristics of CU A- participants in the tracer-specific Flortaucipir and MK-6240 control cohorts

|  | **Flortaucipir CU A– normative cohort (N=290)** | **n** |
| --- | --- | --- |
| Age (years), mean (SD) | 69.6 (6.0) | 290 |
| Education (years), mean (SD) | 16.7 (2.4) | 290 |
| Baseline MMSE, mean (SD) | 29.1 (1.2) | 285 |
| Follow-up (months) | 0 | 290 |
| APOE4 carrier-positive, n (%) | 68 (26) | 261 |
| Female sex, n (%) | 174 (60) | 290 |
| Baseline CDR score, n (%) |  | 284 |
| 0 | 275 (97) |  |
| 0.5 | 9 (3) |  |
| 1 | 0 |  |
|  | **MK-6240 CU A– normative cohort (N=237)** | n |
| Age (years), mean (SD) | 68.6 (7.9) | 237 |
| Education (years), mean (SD) | 14.9 (3.0) | 13 |
| Baseline MMSE, mean (SD) | 29.2 (1.7) | 215 |
| Follow-up (months) | 0 | 237 |
| APOE4 carrier-positive, n (%) | 4 (31) | 13 |
| Female sex, n (%) | 144 (61) | 237 |
| Baseline CDR score, n (%) |  | 160 |
| 0 | 143 (89) |  |
| 0.5 | 17 (11) |  |

A–, amyloid-negative; APOE4, Apolipoprotein E epsilon 4; CDR, clinical dementia rating; CU, cognitively unimpaired; MMSE, mini-mental state examination; SD, standard deviation.

## Supplement table 2. Outputs controlling for age and sex as covariates

| **Figure** | **Region** | **Amyloid/tau status** | **Tracer** | **Comparison** | **Delta** | **SE** | **t** | **p-value** | **Lower  95% CI** | **Upper  95% CI** |
| --- | --- | --- | --- | --- | --- | --- | --- | --- | --- | --- |
| 3A | cortex | A+ | T807 | CU A+ vs CU A- | 0.0170 | 0.0051 | 3.3403 | 0.0005 | 0.0086 | inf |
| 3A | cortex | A+ | T807 | MCI A+ vs CU A- | 0.0295 | 0.0053 | 5.5909 | <0.00001 | 0.0208 | inf |
| 3A | cortex | A+ | T807 | MCI A+ vs CU A+ | 0.0125 | 0.0057 | 2.1893 | 0.0153 | 0.0030 | inf |
| 3B | cortex | A+ | MK-6240 | CU A+ vs CU A- | 0.0190 | 0.0109 | 1.7512 | 0.0412 | 0.0010 | inf |
| 3B | cortex | A+ | MK-6240 | MCI A+ vs CU A- | 0.0321 | 0.0133 | 2.4218 | 0.0086 | 0.0101 | inf |
| 3B | cortex | A+ | MK-6240 | MCI A+ vs CU A+ | 0.0131 | 0.0146 | 0.8954 | 0.1869 | -0.0113 | inf |
| 3C | cortex | A+T+ | T807 | CU A+ vs CU A- | 0.0250 | 0.0076 | 3.3004 | 0.0007 | 0.0124 | inf |
| 3C | cortex | A+T+ | T807 | MCI A+ vs CU A- | 0.0340 | 0.0058 | 5.8739 | <0.00001 | 0.0244 | inf |
| 3C | cortex | A+T+ | T807 | MCI A+ vs CU A+ | 0.0091 | 0.0080 | 1.1251 | 0.1326 | -0.0044 | inf |
| 3D | cortex | A+T+ | MK-6240 | CU A+ vs CU A- | 0.0469 | 0.0135 | 3.4870 | 0.0004 | 0.0246 | inf |
| 3D | cortex | A+T+ | MK-6240 | MCI A+ vs CU A- | 0.0571 | 0.0141 | 4.0355 | 0.0001 | 0.0336 | inf |
| 3D | cortex | A+T+ | MK-6240 | MCI A+ vs CU A+ | 0.0101 | 0.0172 | 0.5887 | 0.2799 | -0.0190 | inf |
| 4A | cortex | A+ | T807 | CU A+ vs CU A- | 0.0233 | 0.0079 | 2.9285 | 0.0019 | 0.0101 | inf |
| 4A | cortex | A+ | T807 | MCI A+ vs CU A- | 0.0277 | 0.0083 | 3.3500 | 0.0005 | 0.0140 | inf |
| 4A | cortex | A+ | T807 | MCI A+ vs CU A+ | 0.0044 | 0.0090 | 0.4907 | 0.3123 | -0.0105 | inf |
| 4B | cortex | A+ | MK-6240 | CU A+ vs CU A- | 0.0152 | 0.0086 | 1.7640 | 0.0401 | 0.0009 | inf |
| 4B | cortex | A+ | MK-6240 | MCI A+ vs CU A- | 0.0233 | 0.0105 | 2.2180 | 0.0143 | 0.0059 | inf |
| 4B | cortex | A+ | MK-6240 | MCI A+ vs CU A+ | 0.0081 | 0.0116 | 0.7007 | 0.2430 | -0.0112 | inf |
| 4C | cortex | A+T+ | T807 | CU A+ vs CU A- | 0.0153 | 0.0098 | 1.5552 | 0.0615 | -0.0010 | inf |
| 4C | cortex | A+T+ | T807 | MCI A+ vs CU A- | 0.0238 | 0.0075 | 3.1645 | 0.0010 | 0.0113 | inf |
| 4C | cortex | A+T+ | T807 | MCI A+ vs CU A+ | 0.0085 | 0.0105 | 0.8157 | 0.2090 | -0.0090 | inf |
| 4D | cortex | A+T+ | MK-6240 | CU A+ vs CU A- | 0.0302 | 0.0108 | 2.7955 | 0.0032 | 0.0122 | inf |
| 4D | cortex | A+T+ | MK-6240 | MCI A+ vs CU A- | 0.0340 | 0.0113 | 2.9920 | 0.0018 | 0.0151 | inf |
| 4D | cortex | A+T+ | MK-6240 | MCI A+ vs CU A+ | 0.0038 | 0.0138 | 0.2724 | 0.3934 | -0.0196 | inf |
| 5A | tau-naïve | A+ | T807 | CU A+ vs CU A- | 0.0120 | 0.0055 | 2.1786 | 0.0154 | 0.0029 | inf |
| 5A | tau-naïve | A+ | T807 | MCI A+ vs CU A- | 0.0187 | 0.0057 | 3.2494 | 0.0007 | 0.0092 | inf |
| 5A | tau-naïve | A+ | T807 | MCI A+ vs CU A+ | 0.0066 | 0.0062 | 1.0623 | 0.1451 | -0.0037 | inf |
| 5B | tau-naïve | A+ | MK-6240 | CU A+ vs CU A- | 0.0167 | 0.0115 | 1.4534 | 0.0743 | -0.0023 | inf |
| 5B | tau-naïve | A+ | MK-6240 | MCI A+ vs CU A- | 0.0583 | 0.0140 | 4.1643 | <0.0001 | 0.0351 | inf |
| 5B | tau-naïve | A+ | MK-6240 | MCI A+ vs CU A+ | 0.0416 | 0.0154 | 2.7002 | 0.0044 | 0.0159 | inf |
| 5C | tau-naïve | A+T+ | T807 | CU A+ vs CU A- | 0.0163 | 0.0070 | 2.3257 | 0.0110 | 0.0047 | inf |
| 5C | tau-naïve | A+T+ | T807 | MCI A+ vs CU A- | 0.0234 | 0.0054 | 4.3600 | <0.0001 | 0.0145 | inf |
| 5C | tau-naïve | A+T+ | T807 | MCI A+ vs CU A+ | 0.0071 | 0.0075 | 0.9518 | 0.1726 | -0.0054 | inf |
| 5D | tau-naïve | A+T+ | MK-6240 | CU A+ vs CU A- | 0.0501 | 0.0149 | 3.3598 | 0.0006 | 0.0253 | inf |
| 5D | tau-naïve | A+T+ | MK-6240 | MCI A+ vs CU A- | 0.0962 | 0.0157 | 6.1370 | <0.00001 | 0.0701 | inf |
| 5D | tau-naïve | A+T+ | MK-6240 | MCI A+ vs CU A+ | 0.0461 | 0.0191 | 2.4128 | 0.0105 | 0.0138 | inf |

A+, amyloid positive; A-, amyloid negative; CI, confidence interval; CU, cognitively unimpaired; inf, infinity; MCI, mildly cognitively impaired; T807, ^18^F-T807; SE, standard error; t, t-statistic; T+, tau positive; T-, tau negative; vs, versus
